# Co-infection of fasciolosis and hydatidosis in cattle at Jimma Municipal Abattoir, Ethiopia: Prevalence, risk factors, and economic impact

**DOI:** 10.1007/s00436-026-08666-6

**Published:** 2026-03-20

**Authors:** Gelan Dule Dahesa, Isayas Asefa Kebede, Abraham Adugna Tilahun, Bizunesh Mideksa Borena, Yeros Jifara Muleta, Haben Fesseha Gebremeskel, Sagni Ragassa Wadajo, Geremew Haile Lemu, Segni Bedasa Gudina

**Affiliations:** 1 Department of Animal Health, Oromia Job Creation and Vocational Bureau of Gedo, TVET College, Gedo, Ethiopia; 2 School of Veterinary Medicine, Ambo University, P.O. Box 19, Guder, Ethiopia; 3 School of Veterinary Medicine, Wolaita Sodo University, Wolaita Sodo, Ethiopia; 4 School of Veterinary Medicine, College of Agricultural Science, Bule Hora University, P.O. Box 144, Bule Hora, Ethiopia; 5 School of Veterinary Medicine, Dambi Dollo University, P.O. Box 260, Dambi Dollo, Ethiopia; 6 College of Veterinary Medicine and Agriculture, Addis Ababa University, Bishoftu, Ethiopia

**Keywords:** Cattle, Co-infection, Echinococcosis, Economic loss, Fasciolosis, Jimma, Prevalence

## Abstract

Parasitic infections of slaughtered animals have a significant public health importance and cause great economic losses. Zoonotic helminths are commonly transmitted to humans through the consumption of raw or undercooked meat of infected animals. However, information on co-infections of major zoonotic parasites in cattle is limited in the study area. Abattoirs are vital for gathering information on zoonotic animal diseases and protecting the public from consuming infected or unhygienic meat. A cross-sectional abattoir study was conducted from November 2023 to July 2024 to estimate the prevalence of co-infections, associated risk factors, and economic losses due to infected organ condemnations in cattle at Jimma Municipal Abattoir, Oromia, Ethiopia. Simple random sampling was used to select the study animals. Prevalence was determined based on records of parasitic infections detected through postmortem examination findings. A total of 384 animals were examined, with an overall co-infection prevalence of 14.3% (95% CI: 11—18); hydatidosis was the most prevalent, followed by fascioliasis at (17.1%, 95% CI: 14—21) and (14.3%, 95% CI: 11—18), respectively. Similarly, the prevalence of *Fasciola hepatica* was 4.4% (95% CI: 2.7—7.1), followed by *F. gigantica* at 1.8% (95% CI: 0.9—3.8). Cyst fertility assessment of 66 cysts showed 20 viable, along with 18 sterile, 8 calcified, 11 non-viable, and 9 mixed (non-viable and viable) cysts. Males had twice the odds of co-infection than females, but this association was not statistically significant (OR = 1.67, 95% CI: 0.57—4.91). The total monetary loss due to co-infections, in terms of organ condemnation, was estimated at 54,000,000 ETB (approximately 432,000 USD (using 1 USD = 125 ETB). No assessed risk factors (sex, age, body condition score (BCS), breed, and origin) showed a statistically significant association with co-infection status. This study revealed the presence of co-infections of fasciolosis and echinococcosis in cattle in the study area, which contribute to significant economic losses. Therefore, appropriate control and prevention measures should be strengthened to reduce infection and associated losses.

## Introduction

Zoonotic diseases, which are transmitted naturally between vertebrate animals and humans, remain a major concern in both veterinary and public health sectors (Rahman et al. 2020). These infections significantly impact societal well-being across the globe, yet many cases remain undocumented and are often underrecognized in disease surveillance systems (Abrahim et al. 2024).

While animals typically serve as the primary reservoirs for these pathogens, human exposure can occur due to various behavioral and environmental risk factors (Rashid et al. 2019; Ola-Fadunsin et al. 2020; Kebede et al. 2025). Among zoonotic pathogens, parasitic infections have gained growing attention in recent years, particularly due to their potential role in opportunistic infections affecting vulnerable populations (Komba 2017).

Parasitic infections in slaughtered animals hold substantial public health significance and are associated with considerable economic losses, often reflecting the conditions under which the animals were reared (Ola-Fadunsin et al. 2020; Kebede et al. 2025). The parasites of greatest concern during meat inspection are typically those with zoonotic potential, particularly those transmissible to humans through the consumption of undercooked or raw meat from infected animals (Gupta et al. 2017). In contrast, some parasites that do not pose a direct zoonotic threat may still render meat and organs aesthetically unacceptable or unsuitable for market due to visible lesions or tissue damage (Rashid et al. 2019). Human infections with serious meat-borne zoonotic parasites, such as cysticercosis and hydatidosis, can result in severe health outcomes, including epilepsy, anaphylactic reactions, halazone disease, and central nervous system complications (Abd Elaziz et al. 2021).

Bovine fasciolosis is a parasitic disease of significant economic importance in cattle, caused by trematodes of the genus *Fasciola* within the family Fasciolidae (Moazeni and Ahmadi 2016). The two major species responsible are *F. hepatica* and *F. gigantica* (Abunna et al. 2010). Fasciolosis leads to substantial economic losses in the livestock sector, primarily through liver condemnation at slaughter and by compromising the animal’s overall immune function (Yalew et al. 2017; Hansh et al. 2023).

Cystic echinococcosis (CE) is a major helminthic zoonosis caused by the larval stage of *Echinococcus granulosus*, a taeniid tapeworm with global distribution (FAO 2003). Its life cycle requires two mammalian hosts: carnivores as definitive hosts and herbivores as intermediate hosts (Woolsey and Miller 2021). In the domestic cycle, dogs act as definitive hosts, while livestock serve as intermediate hosts. The larval form, known as the hydatid cyst, develops in the internal organs of the intermediate host following ingestion of parasite eggs (Woolsey and Miller 2021; Fesseha and Asefa 2022).

Hydatidosis is a major parasitic disease of domestic animals and a significant zoonotic concern, leading to substantial economic losses and public health challenges worldwide (Assefa and Tesfay 2014). Hydatid cysts behave like tumors, disrupting the function of affected organs, impairing growth, reducing milk and meat production, and resulting in organ rejection during meat inspection (Dyab et al. 2017).

Moreover, these diseases pose significant public health and economic challenges, as humans can become infected through accidental ingestion of parasite eggs or larvae shed into the environment via feces from definitive hosts (Berhe et al. 2010). Therefore, helminth control should be prioritized within poverty reduction strategies to improve livestock productivity and help address current and future food security challenges (Krecek and Waller 2006; Sargison 2020; Gilleard et al. 2021; Rehman and Abidi 2022).

Although epidemiological studies on fasciolosis **(**Tolosa and Tigre 2007; Demssie et al. 2012) and hydatidosis **(**Kumsa and Mohammedzein 2014**)** have been conducted separately in and around the study area, data on the co-occurrence of these parasites in cattle from Jimma are currently lacking**,** and no integrated studies have examined their co-occurrence in slaughtered cattle. Furthermore, updated and comprehensive data on the combined prevalence of these diseases are scarce. This gap is particularly important given the local cultural practice of consuming raw or undercooked meat, which increases the risk of zoonotic transmission to humans. Understanding the prevalence and potential co-infection dynamics of fasciolosis and hydatidosis is essential for designing effective control strategies that address both animal health and public health concerns, as well as mitigating economic losses in the livestock sector.

This study, therefore, aimed to estimate the prevalence, associated risk factors, and economic impact of fasciolosis and hydatidosis co-infection in cattle slaughtered at Jimma Municipal Abattoir.

## Materials and methods

### Study area

**Fig. 1:**
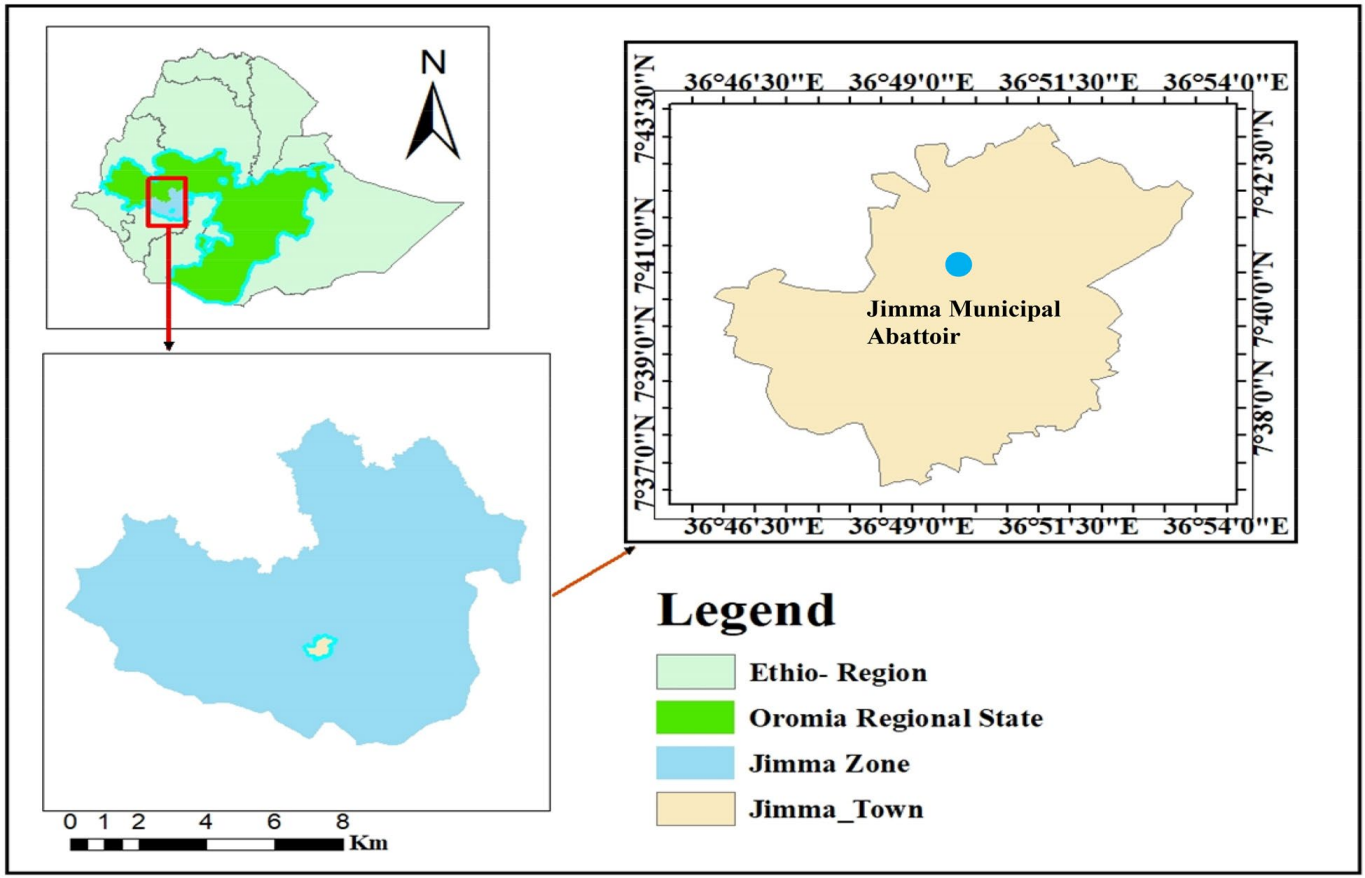
Map of the study area showing the location of Jimma Zone in Ethiopia

The study was conducted at the Jimma town Municipal Abattoir in the Jimma Zone, southwestern Ethiopia (Fig. 1). The Jimma Zone has a total population of approximately 2,495,795, with 94.3% residing in rural areas (Central Statistical Agency of Ethiopia (CSA) 2017). The production system in the zone is predominantly mixed crop-livestock. Jimma is characterized as a highland, moisture-reliable area. Jimma Town, one of the oldest and most historic towns in Ethiopia, is located 352 km southwest of the capital, Addis Ababa (Tsega et al. 2015). Geographically, the town lies between approximately 7°13′ and 8°56′ N latitude and 35°52′ and 37°37′ E longitude, with elevations ranging from 880 to 3,360 m above sea level. The study area receives an average annual rainfall of about 1,530 mm, delivered during both long and short rainy seasons. The mean annual minimum and maximum temperatures are 14.4 °C and 26.7 °C, respectively. The zone experiences a prolonged wet season from March to October, receiving consistent precipitation throughout this period. Livestock populations in the area include cattle (2,560,207), small ruminants such as sheep (859,914) and goats (570,387), apiculture (532,728), poultry (2,235,702), and equines (281,113) (Abagero et al. 2023).

### Study population

The study population consisted of cattle presented for slaughter and routine meat inspection at the Jimma Municipal Abattoir. These animals were brought by traders from Jimma, the surrounding areas, and other districts for slaughter. Depending on their place of origin, the cattle were transported to the abattoir either by truck or on foot. Most of the cattle slaughtered at Jimma Municipal Abattoir originate from the districts of Dedo, Kerisa, and Seka.

During the antemortem inspection, the animals’ age, sex, breed, origin, and BCS were assessed. Cattle were classified into two age groups: adult (≤ 6 years) and old (> 6 years), based on tooth eruption patterns and biological maturity (Yalew et al. 2017). Additionally, BCS were categorized as poor (score 1–3), medium (score 4–6), and good (score > 6), based on observation of anatomical features such as the vertebral column, ribs, and spines (Nicholson and Butterworth 1986).

### Study design

A cross-sectional abattoir-based study was conducted from November 2023 to July 2024 to estimate the prevalence of fasciolosis and cystic echinococcosis in slaughtered cattle at Jimma Municipal Abattoir, Oromia, Ethiopia. Information on the animals’ age, sex, origin, and BCS was recorded during the pre-slaughter inspection.

### Sample size determination and sampling techniques

The sample size was calculated using the formula provided by Thrusfield (2018), with a 95% confidence level and a 5% margin of error.

$$=\frac{{Z}^{2}\times {P}_{exp}\left(1-{P}_{exp}\right)}{{d}^{2}}$$

where: *n* = required sample size; Pexp = expected prevalence (*P* = 50% chosen due to the lack of prior co-infection data in the study area); d = desired absolute precision. Z = 1.96 for a 95% confidence interval. n = 1.962 × 0.5(1–0.5)/0.052 = 3.84 × 0.25/0.0025 = 384.

In the absence of prior data on co-infection prevalence in the study area, a conservative estimate of 50% was used to maximize the required sample size. Using this approach, the calculated sample size was 384 cattle, which were selected using a simple random sampling method.

### Abattoir survey

Antemortem and postmortem examinations were conducted on each sampled animal. During the antemortem inspection, each animal was assigned an identification number, and data on age, sex, breed, origin, and BCS were recorded. For this study, adult and older cattle (Yalew et al. 2017) with medium and good BCS were sampled (Nicholson and Butterworth 1986).

Following this, postmortem examination was performed according to Engdaw et al. (2015) and Kebede et al. (2009). The procedure involved palpation, multiple incisions, inspection of the bile duct, and observation for any irregularities in liver morphology. The presence of *Fasciola* species was carefully assessed. Hydatid cysts were removed from the lungs and liver and examined macroscopically to describe their shape and characteristics.

### Examination of cysts and viability of protoscolices

Each organ’s cysts were counted and inspected. Individual cysts were examined grossly for signs of degeneration or calcification before being transported in an icebox to the Jimma University College of Veterinary Medicine Parasitology Laboratory for fertility and viability testing. Protoscolices from the hydatid fluid were examined microscopically. Following the method described by the FAO (2003), cysts were classified as fertile or infertile; those lacking protoscolices were considered infertile. Viability was assessed by adding a drop of 0.1% aqueous eosin solution to protoscolices on a slide. Live protoscolices exclude the dye partially or completely, while dead ones absorb it. Examination under a 40 × microscope allowed determination of protoscolex viability (FAO 2003; Fesseha and Asefa 2022).

### *Fasciola* species identification

Flukes were collected from bile ducts and liver parenchyma and morphologically identified under a stereomicroscope following FAO (2003). Adult *F. hepatica* are smaller (2.5—3.5 cm × 1.0 cm), leaf-shaped, grey-brown, with distinct shoulders and a conical anterior end. Adult *F. gigantica* are larger (up to 7.5 cm × 1.5 cm), more transparent, with barely perceptible shoulders and a short conical anterior. These key morphological criteria were used to distinguish the two species (Taylor et al. 2007).

### Economic loss estimation

The economic losses due to organ condemnation were estimated based on several factors, including the average annual cattle slaughter rate at Jimma Municipal Abattoir, obtained from historical records, and the average retail prices of affected organs (liver and lung) collected from local butchers during the study period. The annual economic loss was calculated using the formula:

$$\mathrm{EL}=\left({S}_{annual}\times {P}_{liver}\times {C}_{liver}\right)+\left({S}_{annual}\times {P}_{lung}+{C}_{lung}\right)$$

*where:*

$$\mathrm{EL}$$=Economic Losses.

${S}_{annual}$=Annual number of cattle slaughtered.

$P$=Average market price of each organ.

$C$=Organ-specific condemnation rate.

Specifically, the annual loss from liver and lung condemnation, respectively, was calculated as follows: Annual Loss from Liver condemnation = (Annual Slaughter Number) × (Prevalence of Fasciolosis) × (Average Price of Liver), and 

$$\begin{array} {l}{Annual Loss from Liver condemnation }=\\ ({Annual Slaughter Number})\\ \times ({Prevalence of Fasciolosis})\\ \times ({Average Price of Liver})., \end{array}$$

Annual Loss from Lung condemnation = (Annual Slaughter Number) × (Prevalence of Hydatidosis in Lungs) × (Average Price of Lung) (Fesseha and Asefa 2022).

The total number of cattle slaughtered per year was estimated assuming 150 cattle were slaughtered weekly, corresponding to 600 per month and 7,200 per year (150 × 4 × 12 = 7,200) (Source: abattoir recorded data of 2024).

All monetary values were calculated in Ethiopian Birr (ETB) and converted to US dollars using the 2024 exchange rate of 1 USD = 125 ETB (Kebede et al. 2025), corresponding to the rate at the time of the study. Therefore, using the exchange rate at the time of the study, a total loss of 54,000,000 ETB corresponds to 432,000 USD.

### Data management and analysis

Data were entered and recorded in a Microsoft Excel 2016 spreadsheet before being analyzed using STATA^@^ software 14.0. The overall prevalence was calculated as the number of cattle co-infected with hydatidosis and fasciolosis divided by the total number of animals examined. Univariable and multivariable logistic regression analyses were used to explore the impact of risk factors (origin, age, sex, breed, and BCS) on co-infection status. Univariable logistic regression was first applied to assess the association of each risk factor with co-infection. Variables with p-values ≤ 0.25 in the univariable analysis were included in the multivariable logistic regression model and analyzed using backward elimination. Odds Ratios (OR) with 95% confidence intervals (CI) not containing 1 and *p*-values < 0.05 were considered statistically significant.

## Results

Out of 384 cattle slaughtered and examined at the Jimma Municipal Abattoir, 14.3% (95% CI: 11.0—18.0) were found to be co-infected with hydatidosis and fasciolosis (Table 1).

**Table 1:** Prevalence of hydatidosis and fasciolosis in cattle slaughtered at Jimma Municipal Abattoir (*N* = 384)

Infection Status	Number of Examined Cattle	Number of Positive Animals	%	95%CI
Fasciolosis	384	55	14.3	11.0—18.0
Hydatidosis	384	66	17.1	14.0—21.1
Co- infection	384	55	14.3	11.0—18.0

NB: *%* Prevalence, *CI* Confidence Interval, *Co-infection* Simultaneous presence of hydatidosis and fasciolosis.

The current study showed that the prevalence of fasciolosis and cystic echinococcosis was higher in male cattle (17.8%; 95% CI: 14.1—22.2) compared to females (11.4%; 95% CI: 4.3—27.1). Similarly, adult cattle exhibited a higher infection rate (17.9%; 95% CI: 13.9—22.7) than older animals (14.9%; 95% CI: 8.7—23.9). Animals with medium BCS were more affected (21.5%; 95% CI: 4.9—30.0) than those with good BCS (15.3%; 95% CI: 11.5—20.1) (Table 2).

**Table 2:** Prevalence of co-infection (hydatidosis and fasciolosis) across risk factors in cattle slaughtered at Jimma Municipal Abattoir (*N* = 384)

Variables	Categories	NE	NPA	Prevalence	95%CI
Sex	Female	35	4	11.4	4.3—27.1
	Male	349	62	17.8	14.1—22.2
Age	Old	88	13	14.87	8.7—23.9
	Adult	256	53	17.9	13.9—22.7
BCS	Good	268	41	15.3	11.5—20.1
	Medium	116	25	21.5	4.9—30.0
Breed	Local	273	43	15.8	10.7—28.9
	Exotic	39	10	15.7	11.9—20.6
	Cross-breed	72	13	25.6	14.2—41.7
Origin	Kefa	11	0	0	-
	Agaro	46	4	8.7	3.3—21.2
	Jimma	33	4	12.1	4.5—8.7
	Sarbo	50	7	14	6.8—26.8
	Dedo	38	7	18.4	8.9—34.2
	Bilida	84	17	20.2	12.9—30.3
	Seka	51	11	17.7	10.1—29.4
	Asendabo	71	16	22.5	14.2—33.8

NB: *NE* Number of Examined, *NPA* Number of Positive Animals for Co-infection, *BCS* Body Condition Score, *CI* Confidence Interval.

Males had 1.67 times higher odds of co-infection than females, but this association was not statistically significant (95% CI includes 1; *p* = 0.35) (Table 3).

**Table 3:** Univariable analysis of risk factors associated with co-infection and their odds ratios in cattle slaughtered at Jimma Municipal Abattoir

Variables	Categories	NE	NPA	OR	95% CI for OR	p-value
Sex	Female	35	4	Ref	-	-
	Male	349	62	1.67	0.57—4.91	0.35
Age	Old	88	13	Ref	-	-
	Adult	256	53	1.26	0.65—2.43	0.5
BCS	Good	268	41	Ref	-	-
	Medium	116	25	1.52	0.87—2.65	0.12
Breed	Local	273	43	Ref	-	-
	Exotic	39	10	0.85	0.43—1.68	0.65
	Cross-breed	72	13	1.56	0.61—3.99	0.35
Origin	Kefa	11	0	Ref	-	-
	Agaro	46	4	1.45	0.33—6.26	0.62
	Jimma	33	4	1.71	0.47—6.27	0.42
	Sarbo	50	7	2.37	0.64—8.81	0.19
	Dedo	38	7	2.67	0.84—8.46	0.09
	Bilida	84	17	2.26	0.67—7.63	0.19
	Seka	51	11	3.05	0.95—9.81	0.06

NB: *NE* Number of Examined, *NPA* Number of Positive Animals for Co-infection, *BCS* Body Condition Score, *CI* Confidence Interval, *OR* Odds Ratio.

Variables with *p* ≤ 0.25 in the univariable analysis, including BCS and origin, were included in the multivariable logistic regression model to assess their association with co-infection status. However, they were insignificant (Table 4).

**Table 4:** Multivariable logistic regression analysis of risk factors associated with co-infection in cattle slaughtered at Jimma Municipal Abattoir

Variable	Category	Adjusted OR	95% CI for OR	p-value
BCS	Good	Ref	-	-
	Medium	1.4	0.8—2.5	0.12
Origin	Kefa	Ref	-	-
	Dedo	2.5	0.8—8.0	0.09
	Seka	3.0	0.9—9.5	0.06

NB: *Ref* reference category, *OR* Odds Ratio, *CI* Confidence Interval.

**Fig. 2:**
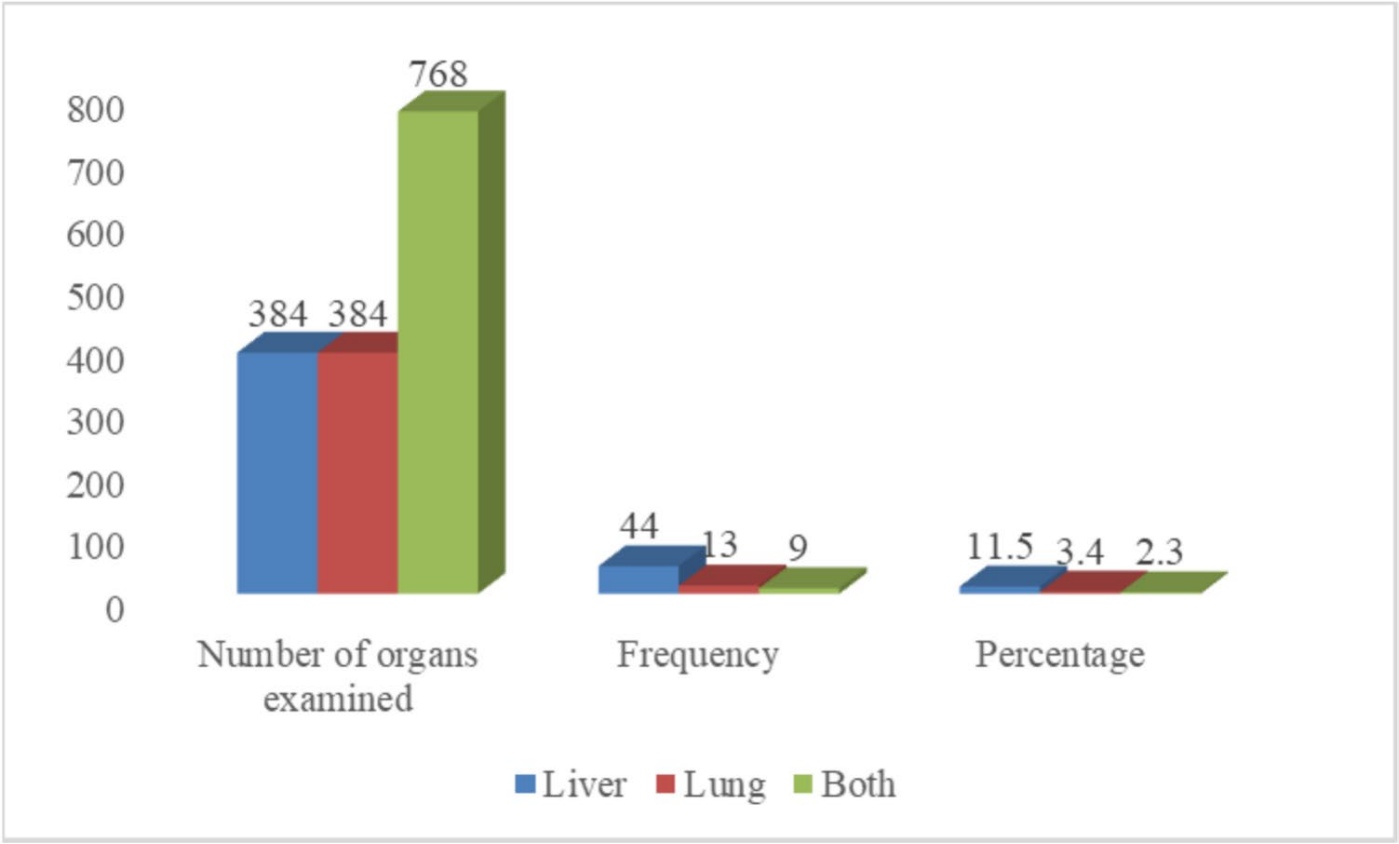
Prevalence of hydatidosis by organ site in cattle slaughtered at Jimma Municipal Abattoir. NB: Both: Liver and Lung concurrently

Out of 384 cattle slaughtered and examined at Jimma Municipal Abattoir, 66 (17.1%) were positive for hydatidosis. Organ-specific prevalence was 11.5% in the liver, 3.4% in the lungs, and 2.3% in both organs (Fig. 2).

For fertility analysis, only one cyst per positive animal was examined, resulting in 66 cysts analyzed for viability and sterility. Among these, 20 cysts were viable, and 18 were sterile (Table 5). This approach was taken due to time and economic constraints, and the study was conducted without external funding.

**Table 5:** Distribution of hydatid cysts in the lung and liver according to fertility status in cattle slaughtered at Jimma Municipal Abattoir

Organ	Types of cysts					Total
	Viable n (%)	Non-Viable n (%)	Mixed n (%)	Sterile cyst n (%)	Calcified cyst n (%)	n (%)
Liver	16	8	7	11	7	49 (74.2)
Lung	4	3	2	7	1	17 (25.8)
Total	20 (30.3)	11 (16.7)	9 (13.6)	18 (27.3)	8 (12.1)	66

NB: *n* Frequency, *%* Percentage.

**Fig. 3:**
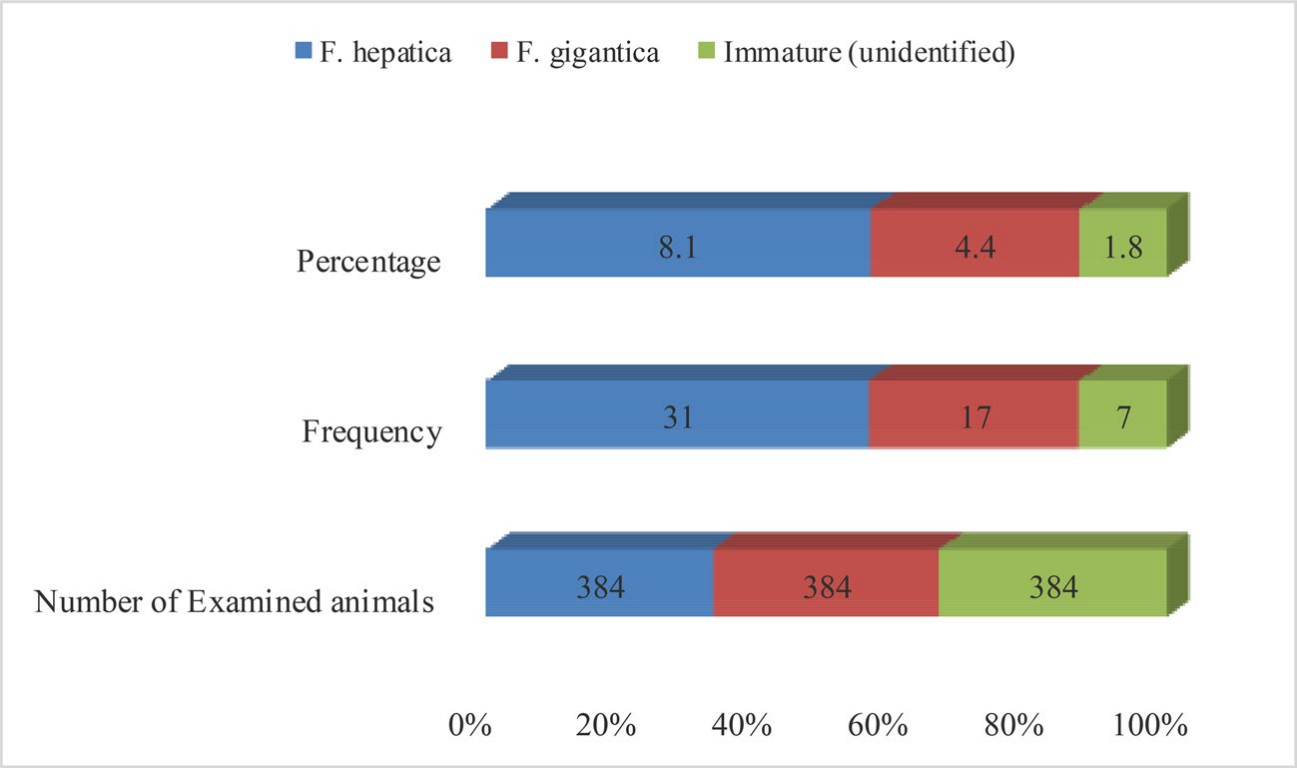
Prevalence of liver fluke infections in cattle at Jimma Municipal Abattoir (*n* = 384): *F. hepatica* 8.1%, *F. gigantica* 4.4%, and immature/unidentified flukes 1.8% (overall fasciolosis prevalence 14.3%)

Out of 55 cattle positive for liver fluke infection during postmortem inspection, 8.1% (95% CI: 5.7—11.3) were infected with *F. hepatica* (Fig. 3).

### Direct economic loss due to co-infection of hydatidosis and fasciolosis

The direct annual economic loss caused by fasciolosis and hydatidosis was calculated using the formula: Yearly economic loss = Annual Loss from Liver Condemnation = ((Annual Slaughter Number) × (Prevalence of Fasciolosis) × (Average Price of Liver)) + Annual Loss from Lung Condemnation = ((Annual Slaughter Number) × (Prevalence of Hydatidosis in Lungs) × (Average Price of Lung)). The total number of cattle slaughtered per year was estimated assuming 150 cattle slaughtered weekly, corresponding to 600 per month and 7,200 per year (150 × 4 × 12 = 7,200) (Table 6). Based on this, the annual economic loss was calculated as (14.3 × 375 × 7,200) + (17.1 × 125 × 7,200) = 54,000,000 ETB or approximately 432,000 USD.

**Table 6:** Direct economic loss due to condemned liver and lung as a result of the prevalence of co-infection (hydatidosis and fasciolosis) across risk factors in cattle slaughtered at Jimma Municipal Abattoir

Species	AE	Organ type	Total examined organs	No of positive organs	Percent involvement of the organ	Average unit price Total loss (ETB)	Total loss (ETB)	Total loss USD
Bovine	384	Liver	384	55	14.3	375	38,610,000	308,880
		Lung	384	66	17.1	125	15,390,000	123,120
Total			768	121	15.8	500	54,000,000	432,000

NB: *AE* Total number of animals examined, *ETB* Ethiopian Birr

Currency conversion rate: 1 USD = 125 ETB (2024)

Annual slaughter estimated at 7,200 cattle (150/week × 48 weeks)

## Discussion

In the present study, the overall prevalence of co-infection (fasciolosis and hydatidosis) was 14.3% (95% CI: 11.0—18.0). This indicates that co-infection is present at moderate levels among cattle in Jimma. This finding is lower than the 27.34% reported at Al-Nassiriyah City Abattoir, Iraq (Hansh et al. 2023); however, higher than the 4.17% reported from Wolaita Sodo Municipal Abattoir, Southern Ethiopia (Fesseha and Asefa 2022), 3.93% at Butajira Municipal Abattoir in southern Ethiopia (Kerala et al. 2021), and 8.5% at Mekelle Municipal Abattoir, Tigray Region (Berhe et al. 2010). Such variation may be attributed to differences in environmental conditions, husbandry practices, lifestyle and social factors, informal or backyard slaughtering practices, poor offal disposal, and local perceptions regarding stray dogs (Hansh et al. 2023).

### Fasciolosis prevalence

The overall prevalence of fasciolosis in this study was 14.3% (95% CI: 11—18), which is consistent with the 14.0% reported at Soddo Municipal Abattoir, Southern Ethiopia (Abunna et al. 2010). This prevalence is lower than the 35% reported in El-Minia Governorate Abattoirs, Egypt (Dyab et al. 2019), 49.89% Ethiopian abattoir (Molla et al. 2019), and 26.6% in and around Zenzelma, Bahir Dar, Ethiopia (Legesse et al. 2017), but higher than findings from Wolaita Sodo (9.38%) (Fesseha and Asefa 2022), Al-Nassiriyah City, Iraq (7.25%) (Hansh et al. 2023), and the Mid-Delta region of Egypt (0.76%) (Elmonir et al. 2015). Differences in prevalence may be attributed to variations in farmer awareness, implementation of parasite control measures, and environmental risk factors, such as open defecation by animals near water canals, use of contaminated irrigation water, and ingestion of roughages or water containing encysted metacercariae (John et al. 2019; Fesseha and Asefa 2022).

Fasciolosis in this study was predominantly caused by *F. hepatica* (8.1%), followed by *F. gigantica* (4.4%) and unidentified immature flukes (1.8%). The prevalence of *F. hepatica* is similar to 8.59% reported in Wolaita Sodo Municipal Abattoir (Fesseha and Asefa 2022), but higher than 3.70% reported by Leka (2019) and 2.47% in a mechanical abattoir (Rahman et al. 2020). *F. gigantica* prevalence (4.4%) aligns with 4.175% reported in Wolaita Sodo (Fesseha and Asefa 2022), but is lower than 22.2% reported by Leka (2019) and higher than 1.09% in a mechanical abattoir (Rahman et al. 2020). The prevalence of immature flukes (1.8%) is comparable to 0.78% reported by Fesseha and Asefa (2022).

These differences may reflect local climatic and agroecological conditions that affect snail populations and parasite transmission, including the limited availability of suitable snail habitats and the optimal base temperatures required for snail vectors—approximately 10 °C for *F. hepatica* and 16 °C for *F. gigantica*. Local climate, access to permanent water bodies, and management practices may also influence disease prevalence and severity across regions (Leka 2019).

### Hydatidosis prevalence

The prevalence of cystic echinococcosis (CE) was 17.9% (95% CI: 14—21), consistent with 18.61% reported at Adigrat Municipal Abattoir, Ethiopia (Assefa and Tesfay 2014) and 16.66% in Ethiopian abattoirs (Molla et al. 2019). This is higher than 8.40% reported in Al-Nassiriyah City, Iraq (Hansh et al. 2023), 5.3% in Hamadan Abattoir, Western Iran (Roostaei et al. 2017), and 9.4% in Turkey (Balkaya and Simsek 2010). Variation in prevalence across regions may be influenced by local dog ownership practices, including feeding infected offal to dogs, which facilitates pasture contamination with Echinococcus eggs. Backyard slaughtering, improper disposal of infected organs, poor control of stray dogs, and inadequate hygienic conditions in abattoirs may also increase transmission. Additionally, differences in livestock management and the close association of animals with dogs can affect the occurrence of hydatidosis (Assefa and Tesfay 2014; Leka 2019).

Hydatid cysts were most frequently found in the liver at 11.5% (95% CI: 8.6—15), followed by the lungs at 3.4% (95% CI: 1.9—5.8), and both organs concurrently at 2.3% (95% CI: 1.2—4.5). Among 66 cysts examined for fertility, 20 were viable, 11 non-viable, 18 sterile, 8 calcified, and 9 mixed (both viable and non-viable) cysts. These findings align with previous reports from Wolaita Sodo Municipal Abattoir, Southern Ethiopia (Fesseha and Asefa 2022), Mekelle Municipal Abattoir, Tigray Region (Berhe et al. 2010), and Jimma, Southwestern Oromia (Kumsa and Mohammedzein 2014). The liver and lungs are more frequently affected because their dense capillary networks act as the first filters for oncospheres migrating from the gut through the portal circulation (Kumsa and Mohammedzein 2014).

### Associated risk factors

Adult cattle showed a higher observed prevalence of fasciolosis and cystic echinococcosis (1.26%, OR: 0.65–2.43), but differences among age groups were not statistically significant (*p* = 0.5). This is consistent with reports from Mekelle Municipal Abattoir, Tigray Region (Berhe et al. 2010), and Wolaita Sodo Municipal Abattoir, Southern Ethiopia (Fesseha and Asefa 2022). The slight decrease in infection with increasing age may reflect acquired immunity over time, characterized by humoral immune responses and tissue reactions following prior exposure (Assefa and Tesfay 2014).

The highest prevalence was observed in cattle with medium BCS (21.5%; 95% CI: 4.9—30.0), followed by those in good condition (15.3%; 95% CI: 11.5—20.1). Although animals with poor BCS are generally more susceptible to chronic parasitic infections (Muhammed and Birhanu 2020), this study did not find a statistically significant difference in BCS.

Cattle origin showed some variation in odds of co-infection, with animals from Seka having the highest odds (OR = 3.05; 95% CI: 0.95—9.81), followed by Dedo (OR = 2.67; 95% CI: 0.84—8.46) and Sarbo (OR = 2.37; 95% CI: 0.64—8.81) compared to Kefa. However, these differences were not statistically significant (*p* > 0.05), suggesting that origin alone was not a strong predictor of co-infection. Local environmental or management variations may contribute, such as the availability of snail habitats favorable for *Fasciola* transmission.

No significant associations were found between co-infection and sex, age, BCS, breed, or origin. This finding aligns with previous reports on breed (Muhammed and Birhanu 2020; Kebede et al. 2025), BCS (Berhe et al. 2010; Fesseha and Asefa 2022; Kebede et al. 2025), and age (Fesseha and Asefa 2022; Kebede et al. 2025). The lack of significance may be due to homogeneous exposure across the study population, uniform management and treatment practices, and a relatively limited sample size. For instance, males and females were managed similarly and had equal access to potentially contaminated pastures, which may explain why sex was not associated with co-infection. Likewise, breed differences were not significant, possibly due to unknown or variable levels of cross-breeding (Hansh et al. 2023). Variables with p ≥ 0.25 in the univariable analysis (sex, age, and breed) were excluded from the final multivariable model to focus on predictors with potential associations and improve model fit.

Generally, these findings indicate that co-infection may be influenced by complex or unmeasured factors beyond the variables examined in this study, highlighting the need for further research on local management, environmental, and host-related determinants.

### Economic loss

The total direct monetary loss due to co-infection, considering organ condemnation and carcass weight, was estimated at 54,000,000 ETB (approximately 432,000 USD). This exceeds 16,800.4 USD reported by Elmonir et al. (2015) but is lower than the 11.7 billion USD annual loss reported in Saudi Arabia due to fascioliasis (Degheidy and Abd Elaziz 2021). It is also higher than 15,436,142 ETB recorded at Wolaita Sodo Municipal Abattoir (Fesseha and Asefa 2022). These figures represent direct economic losses and do not account for indirect costs such as reduced weight gain, milk production, fertility, or treatment expenses. Differences in economic losses across regions largely reflect variations in disease prevalence and local market prices.

### Public health implications

The high incidence of these parasitic infections indicates insufficient control measures, limited anthelmintic coverage, and low public awareness. Cultural practices, such as consumption of raw or undercooked beef liver (‘*kourt’*, ‘*lebleb’*, ‘*kitffo’*), and open defecation, contribute to environmental contamination and zoonotic transmission (Molla et al. 2019). Humans may acquire infection through the ingestion of metacercaria on aquatic plants or vegetables.

Therefore, appropriate control measures should be implemented, including raising public awareness, proper drainage of marshy areas, clearing aquatic vegetation, safe disposal of condemned offal or meat, minimizing pasture contamination, and regular treatment of animals with broad-spectrum anthelmintics. Given that hydatidosis and fascioliasis are zoonotic, with hydatidosis ranked fifth (after rabies, anthrax, brucellosis, and leptospirosis) among 43 prioritized zoonotic diseases in Ethiopia (Pieracci et al. 2016) and several human fasciolosis cases reported in the country (Aregahagn and Asrat 2018), effective control requires a One Health approach. This approach should involve interdisciplinary collaboration among veterinary and human health professionals and environmentalists to reduce both animal and human health risks (Pieracci et al. 2016; Kebede et al. 2025).

### Limitations of the study

This study has some limitations. First, the cross-sectional design captures a snapshot, limiting inference on causality and seasonal variation. Second, reliance on abattoir data may not reflect community-level prevalence, and data were collected from a single abattoir, which may not represent the entire cattle population in the region. Third, postmortem examination might underestimate true prevalence due to subclinical infections or missed lesions. Fourth, some important risk factors, such as detailed management practices, environmental variables, and seasonal variation, were not assessed. Finally, the economic estimates account only for direct losses from organ condemnation and do not include indirect costs, such as reduced weight gain, fertility, milk yield, or treatment costs, which are likely substantial. Future research should address these gaps to provide a more comprehensive understanding of co-infection impacts.

## Conclusion

Fasciolosis and hydatidosis remain significant zoonotic parasitic diseases that pose both economic and public health challenges in livestock production. This study identified a 14.3% prevalence of co-infection, with hydatidosis predominating at 17.1% and fascioliasis at 14.3%. Although no statistically significant risk factors were linked to co-infection, the economic impact, estimated at 54 million ETB, underscores the substantial burden on the local cattle industry. These results underscore the urgent need for comprehensive epidemiological investigations tailored to the region’s agroecological context. Effective control strategies should prioritize breaking the life cycle of cystic echinococcosis through integrated interventions, alongside enhancing abattoir sanitation, meat inspection protocols, and reporting systems. Strengthening these measures will be critical to mitigating economic losses and reducing zoonotic transmission risks to humans.

## Data Availability

All the datasets generated or analyzed during this study are included in this manuscript.
